# Transcriptomic analysis reveals mechanism of light-sensitive albinism in tea plant *Camellia sinensis* ‘Huangjinju’

**DOI:** 10.1186/s12870-020-02425-0

**Published:** 2020-05-14

**Authors:** Xinfeng Jiang, Hua Zhao, Fei Guo, Xuping Shi, Chuan Ye, Puxiang Yang, Benying Liu, Dejiang Ni

**Affiliations:** 1 College of Horticulture and Forestry Science, Huazhong Agricultural University, 1 Shizishan Street, Hongshan District, Wuhan, 430070 Hubei China; 2 Jiangxi Sericulture and Tea Research Institute, Nanchang, 330202 Jiangxi China; 3 Yunnan Provincial Key Laboratory of Tea Science, Jinghong, 666100 Yunnan China

**Keywords:** Chloroplast, Leaf albinism, Leaf pigmentation, Metabolites, Photosynthesis, Tea

## Abstract

**Background:**

*Camellia sinensis* ‘Huangjinju’ is an albino tea variety developed recently in China. Young leaves of ‘Huangjinju’ demonstrate bright yellow when cultivated under natural sunlight, but regreens under reduced light intensity. To elucidate the physiological and molecular mechanisms of this light-sensitive albinism, we compared leaf pigmentation, metabolites, cellular ultrastructure and transcriptome between plants cultured under natural sunlight and shade.

**Results:**

Shading treatment doubled the chlorophyll concentration and regreened albino leaves; carotenoid also increased by 30%. Electron microscopy analyses showed that chloroplast not only increased in number but also in size with a complete set of components. In addition, regreened leaves also had a significantly higher concentration of polyphenols and catechins than albino leaves. At transcriptomic level, a total of 507 genes were differentially expressed in response to light condition changes. The most enriched pathways include light harvest protein complex, response to stimuli, oxidation-reduction process, generation of precursor metabolites and energy response.

**Conclusion:**

The integrated strategy in this study allows a mechanistic understanding of leaf albinism in light-sensitive tea plants and suggested the regulation of gene networks involved in pigmentation and protein processing. Results from this study provide valuable information to this area and can benefit the domestication and artificial breeding to develop new albino tea varieties.

## Background

Leaf color is a plastic phenotype and changes under different environmental conditions (e.g., light intensity, temperature and media composition). Leaf albinism is often considered deleterious and not favored in agriculture because it lacks essential pigmentation for normal functions such as photosynthesis [1, 2]; on the other hand, it produces specialties with some unique characteristics. In tea plant, for example, young albino leaves/shoots are commercially grown because of its unique flavor compared to regular green tea [3]. There are two types of albinism in *Camellia sinensis*: temperature-sensitive (e.g., cultivars ‘Anji Baicha’, ‘Baiye 1’ and ‘Xiaoxueya’) and light-sensitive (e.g., cultivars ‘Huangjinju’, ‘Huangjinya’ and ‘Baijiuan’) [4]. As albino tea becomes internationally popular and the market continues to grow, understanding its molecular basis is in high demand and has a great economic value.

Light-sensitive albinism in tea plants involves modification in physiological and biochemical processes such as pigmentation, intracellular structure and metabolites [4]. The early development of leaves in albinistic plant typically experiences three stages: pre-albinistic stage, albinistic stage and regreening stage [5]. Most leaf albinism occurs in the albinistic stage, which lasts from several weeks to months. Reducing light intensity by artificial shielding often regreens leaves to the normal level. In the albinistic stage, leaf color varies from white to yellow depending on the level of deficiency in chlorophyll and carotenoids. Associated with the decrease in pigmentation is the often-observed abnormal development of chloroplasts and thylakoid membranes [6]. Albinism is also coupled with modified biochemical processes that contribute to changes in metabolites [7], which determines the flavor of brewed tea. Specifically, for example, high levels of amino acids bring an ‘umami’ taste; low levels of caffeine and catechins decrease the astringency and bitterness [3, 6, 8].

In recent years, molecular techniques such as transcriptome sequencing provide opportunities to study the molecular mechanisms of tea albinism. Some photosynthesis-related genes and pathways have been shown to be involved in leaf albinism in tea plant, including pigmentation synthesis, protein processing, oxidation-reduction and flavonoid biosynthesis [9]. Pathways that contribute to essential metabolites are also shown to be involved, such as flavonoid biosynthesis and amino acid metabolism [4]. Although some genes and pathways are expected to be universally present in plant albinism, the ones that contribute to unique characteristics of each tea cultivar is of the greatest interest. The majority of existing literature has focused on several major cultivars such as *C. sinensis* ‘Anji Baicha’ [3, 5, 7, 10–12]. Study on new tea varieties that are being actively developed is still limited.

‘Huangjinju’ is a light-sensitive albino cultivar of *C. sinensis* propagated from a natural variant in Jiangxi province, China [13]. Young leaves of ‘Huangjinju’ demonstrate yellow under natural sunlight, but gradually regreen as development progress. This study marks the first effort to explore the albinism mechanism in this cultivar. We applied integrated approaches to examine the response of pigmentation accumulation, metabolites, ultracellular structure and transcriptome to different light conditions by culturing plants under natural sunlight and shade. Results confirmed the role of some genes and pathways in photosynthesis and protein processing, but also identify additional pathways that are regulated to produce the unique characteristics in albino leaves.

## Results

### Leaf pigmentation, chloroplast and metabolites concentration

**Fig. 1:**
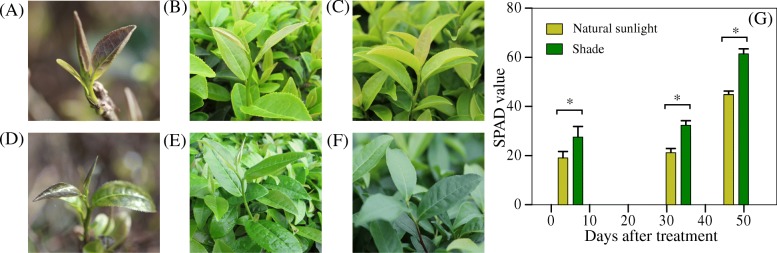
Color differences in *C. sinensis* ‘Huangjinju’ cultured under natural sunlight versus under shade. **a-c** natural sunlight. **d-f** shade treatment. **a** and **d** before treatment. **b** and **e** 20 days after treatment. **c** and **f** 33 days after treatment. **g** a bar plot of SPAD value for leaves under natural sunlight and shade. The asterisks indicate significant differences between treatments (*p* < 0.05). SPAD values were measured in the same day on day 5, 33 and 48 after treatment

Young shoots of ‘Huangjinju’ emerged after trimming were yellow (Fig. 1). After 5 days of shading treatment, pale yellow leaves under shade gradually turned green. To quantify the “greenness” of leaves, we first measured Soil-Plant Analyses Development (SPAD) values in the field. By taking measurements on a total of 60 leaves for each treatment, SPAD values in shaded leaves were 36.8–52.3% higher than that in exposed leaves (Fig. 1g). The concentration of chlorophyll-a and chlorophyll-b in shaded leaves was twice as much as that in leaves under natural sunlight (Table 1), which agreed with the SPAD results. Similarly, carotenoids were also 30% higher in shaded leaves. We also observed that regreened leaves under shade had a significantly higher concentration of polyphenols and catechins than albino leaves under natural sunlight (Table 2).

**Table 1:** Pigment concentration of young shoots in *C. sinensis* ‘Huangjinju’ under natural sunlight (NS) and shade (S)

	Sample mass (g)	Chlorophyll-a (mg/g)	Chlorophyll-b (mg/g)	Total chlorophyll (mg/g)	Carotenoids (mg/g)
NS-1	0.2032	0.40	0.08	0.5	320.3
NS-2	0.2085	0.50	0.10	0.6	373.7
S-1	0.2072	0.97	0.24	1.2	490.8
S-2	0.208	0.83	0.22	1.1	441.8
NS/S	1.0	0.5	0.4	0.5	0.7
S/NS	1.0	2.0	2.6	2.1	1.3

Pigmentation concentration was measured twice for both treatments. Each measurement used 100 randomly harvested leaves. The ratio of the average values between treatments is also calculated

**Table 2:** Concentrations of tea metabolites in *C. sinensis* ‘Huangjinju’ under natural sunlight and shade

	Natural sunlight	Shade
N	8	8
Amino acid	2.09 ± 0.05	2.13 ± 0.05
Caffeine	3.23 ± 0.07	3.41 ± 0.14
Polyphenol	19.8 ± 1.6^a^	24.9 ± 1.3^b^
Total Catechin	15.1 ± 1.5^a^	18.2 ± 1.1^b^
Gallate	0.030 ± 0.007	0.026 ± 0.005
Epigallocatechin	0.22 ± 0.01^a^	0.28 ± 0.02^b^
Catechin	0.08 ± 0.01^a^	0.12 ± 0.01^b^
Epicatechin	0.47 ± 0.07	0.53 ± 0.03
Epigallocatechin gallate	12.2 ± 1.7	14.3 ± 1.0
Gallocatechin gallate	0.18 ± 0.05	0.17 ± 0.04
Epicatechin gallate	1.78 ± 0.4^a^	2.51 ± 0.4^b^
Catechin gallate	0.13 ± 0.01^a^	0.20 ± 0.03^b^

All numbers are relative weight (%). Data are presented as mean ± s.e.m. Significant differences (*p* < 0.05) between treatments are denoted with different lower-case letters

### Cell ultrastructure

**Fig. 2:**
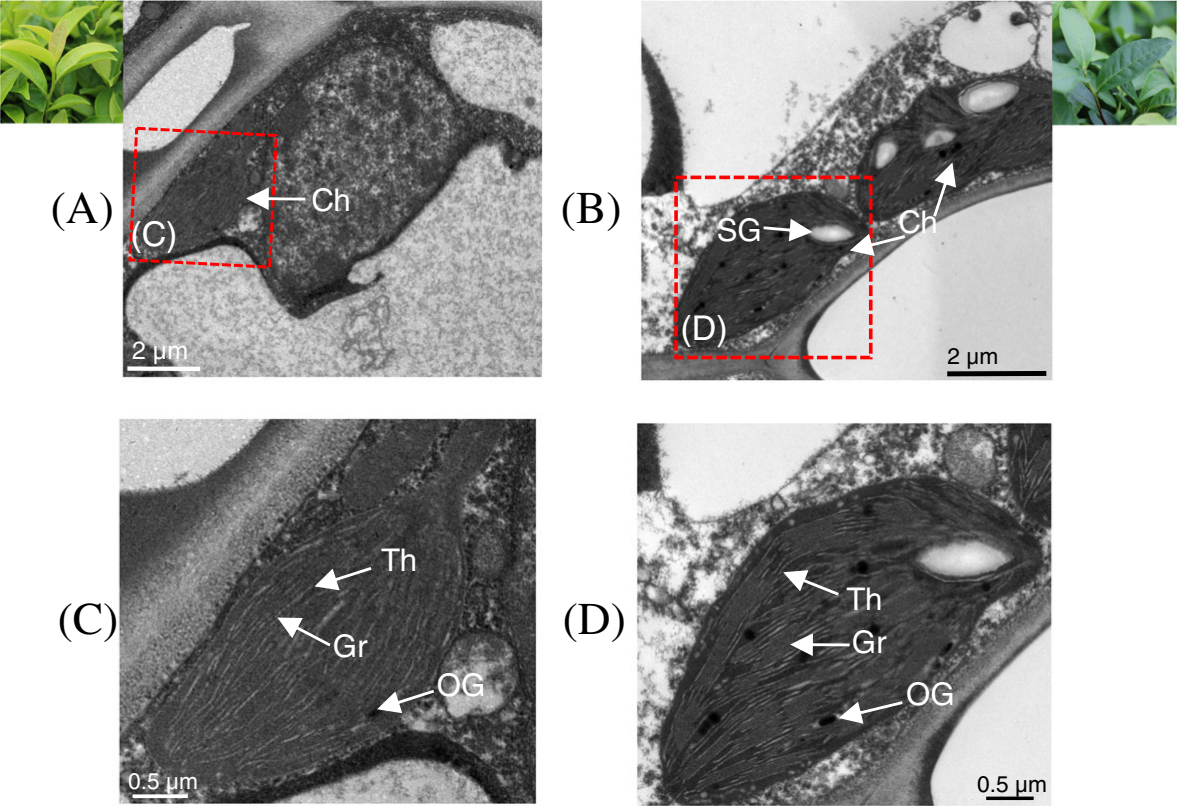
Cellular ultrastructure of young shoots in *C. sinensis* ‘Huangjinju’ under direct sunlight and shade. **a** and **c** leaves under natural sunlight. **b** and **d** leaves under shade. Ch: chloroplast; SG: starch granule; OG: osmiophilic granule; Th: thylakoid; Gr: grana

Transmission electron microscopy (TEM) images showed abnormal cellular ultrastructure in leaves cultured under different light conditions. Chloroplast of leaves under natural sunlight had fewer starch granules (SG), osmiophilic granules (OG), and thylakoids (Th) stacking (Fig. 2a and c). In contrast, chloroplasts were fully developed in shaded leaf and no abnormity was found in thylakoid membranes and granular stacking (Fig. 2b and d).

### Differential gene expression

**Fig. 3:**
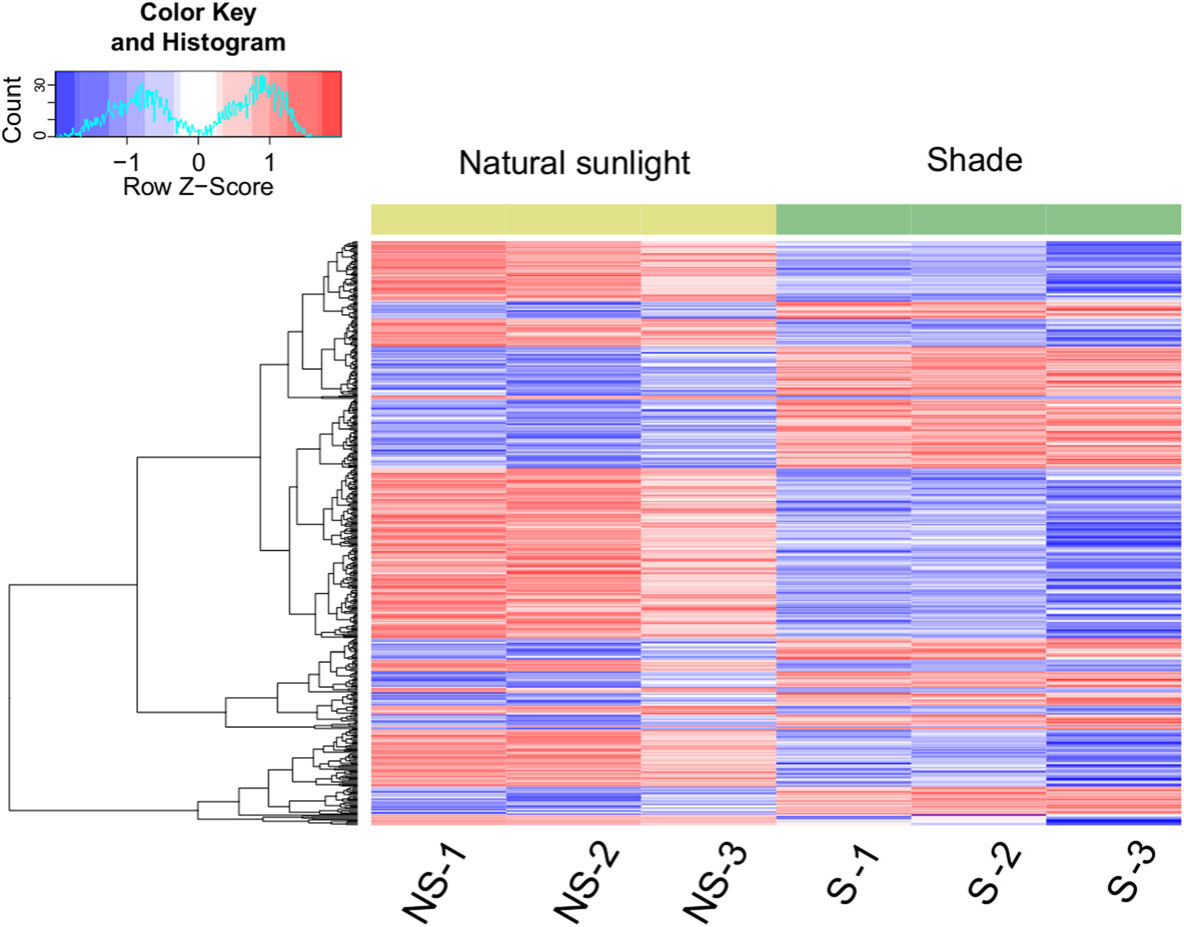
Heatmap of differentially expressed genes in plants under shade (S) versus under natural sunlight (NS)

A total of 304.8 million raw reads were obtained from the sequencer. After applying quality filtering processes, 301.8 million clean reads were retained for downstream analysis. An average of 85.2% of the total reads was uniquely aligned to the reference genome. A total of 41,444 unique transcripts were identified. Compared to plants under natural sunlight, plants under shade treatment significantly regulated 507 genes (Fig. 3), which includes 198 up-regulated genes and 309 down-regulated transcripts (Details of the differentially expressed gene (DEGs) are given in supplementary Table S1). The most significantly up-regulated gene is *WRKY30* (WRKY DNA-binding protein 30) with a 9.4-fold increase, while the most significantly down-regulated DEG is *CYP* (cytochrome P450) with 111-fold decrease. The gene that held the greatest absolute difference is *LHCB* (light-harvesting complex II chlorophyll a-b binding protein) followed by *LIP* (light-inducible protein).

**Fig. 4:**
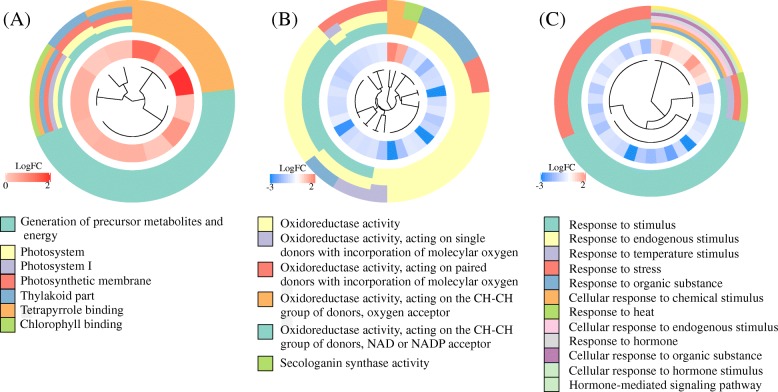
Gene ontology (GO) analysis of differentially expressed genes. Three significant terms and their child terms are selected to present. **a** GO:0006091, “generation of precursor metabolites and energy”. **b** GO:0016491: “oxidoreductase activity”. **c** GO:0050896: “response to stimuli”. Inner circles represent fold change of differentially expressed genes (DEGs) in plants cultured under shade compared to plants cultured under direct sunlight. Outer circles represent the GO terms of DEGs

Gene ontology (GO) analysis suggested that DEGs were mostly involved in three categories: “response to stimulus”, “oxidation-reduction process” and “generation of precursor metabolites and energy response” (Fig. 4 and Fig. S1). The child terms of “response to stimulus” include functions related to “response to heat”, “response to organic substance” and “response to hormone”. Some DEGs in “generation of precursor metabolites and energy response” are involved in photosynthesis processes. Pathways of processing denatured proteins and misfolded proteins became less active as suggested by the downregulation of heat shock protein genes. Functional categories of DEGs from GO analyses are listed in supplementary Table S2.

**Fig. 5:**
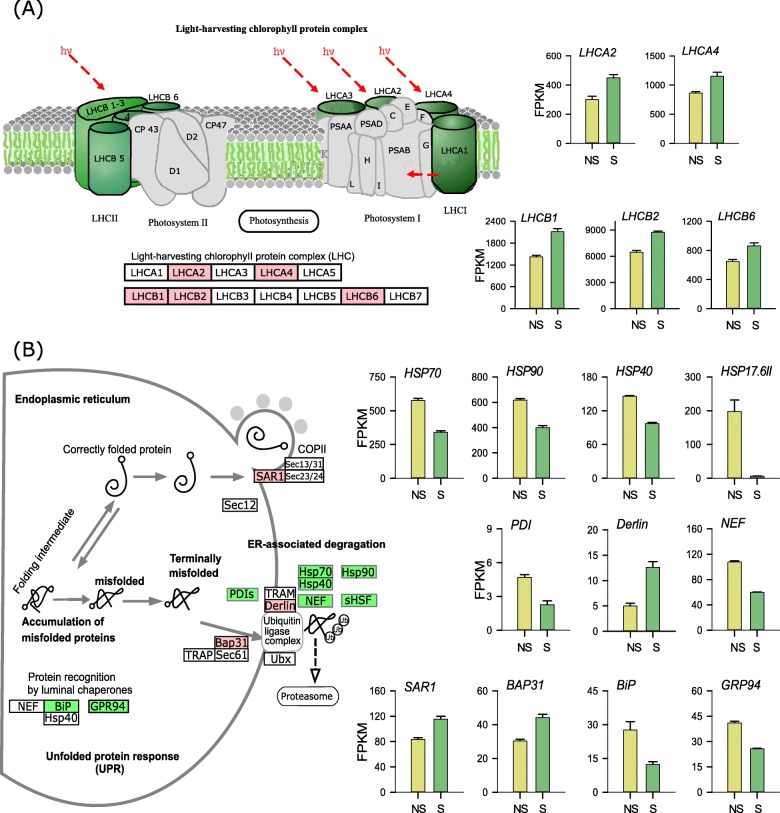
KEGG pathway analysis of differentially expressed genes in *C. sinensis* ‘Huangjinju’ under shade (S) and natural sunlight (NS). **a** Significantly regulated genes in KEGG pathway “Photosynthesis - antenna proteins (ath00196)” and bar plots of gene expression. **b** Significantly regulated genes in KEGG pathway “Protein processing in the endoplasmic reticulum (ath04141)” and bar plots of gene expression. The use of pathway maps (ath00196 and ath04141) was permitted by KEGG (permission 200198). Up-regulated genes are in red background and downregulated genes are in blue background-color. Each bar represents mean + s.e.m.

Mapping to Kyoto Encyclopedia of Genes and Genomes (KEGG) ortholog database revealed three significantly enriched pathways: “Photosynthesis - antenna proteins” (ath00196) (Fig. 5a), “Protein processing in endoplasmic reticulum” (ath04141) (Fig. 5b) and “Brassinosteroid biosynthesis” (ath00905). Some DEGs in “Protein processing in endoplasmic reticulum” pathway was also involved in endocytosis, plant-pathogen interaction, spliceosome and protein export processes. DEGs were also discovered in other pathways such as “Photosynthesis”, “flavonoid biosynthesis pathway”, “Biosynthesis of amino acid pathway” and “Carotenoid biosynthesis”, which are known to be involved in response to light condition changes. Functional categories of DEGs KEGG analyses are listed in supplementary Table S3.

## Discussion

Light intensity is among the most critical environmental factors affecting plant physiology and biochemistry. In most situations, albinism is a hereditary condition caused by mutations that may have occurred in the nuclear or chloroplast genomes. The molecular mechanism likely not only involves a group of genes but also some degree of interaction among them. Here we show that albino tea plants significantly regulated the expression of numerous structural and regulatory genes in response to light. Reducing light intensity causes the pale yellow ‘Huangjinju’ leaves to regreen with increased chlorophyll and carotenoid deposition. Regreened leaves also had significantly higher polyphenols and catechins content than pale leaves. Meanwhile, the gene expression profile significantly differed between treatments in pathways such as photosynthesis, protein folding, amino acid metabolism, cellular structure and oxidation.

Leaf color variation is determined by pigmentations, including chlorophyll and carotenoid. Albino ‘Huangjinju’ leaves under natural sunlight are low in chlorophyll, therefore, lack the green pigmentation. Leaves also demonstrated some yellow color, despite that carotenoid was decreased by 20%. Lack of chlorophyll accumulation is a result of the maldevelopment of chloroplast [14]. Aberrant chloroplast has been found to be universal in both light-sensitive and temperature-sensitive albino cultivars. Direct exposure to natural sunlight induces hypoplasia of chloroplasts in young shoots by suppressing the development of grana stacking and thylakoids.

### Photosynthesis pathway

The light-harvesting complex is an aggregate of proteins and photosensitive pigments that absorb light and transfer energy. The contents of pigment–protein complexes gradually increase during the regreening stages in albino plants [10]. We observed significant upregulation of transcripts from chlorophyll a-b binding protein genes in regreened leaves under the shade, suggesting the recovery of photosynthesis activities. Antenna complex also contains carotenoids, lutein, violaxanthin and b-carotene. Beta-carotene isomerase gene was downregulated, which reduced the digestion of β-carotene and helped to accumulate β-carotene. We also observed the downregulation of 9-cis-epoxycarotenoid dioxygenase that catalyzes Violaxanthin / Neoxanthin to Xanthoxin. Xanthoxin is a precursor of abscisic acid that is important for plants to deal with environmental stressors. This result is suggesting that the stress level of plants under reduced light intensity has decreased. It is a similar result as that in *C. sinensis* ‘Huangjinya’, where carotenoid biosynthesis-related genes were induced by shading treatment [15]. We also observed the upregulation of light-responsive gene *PORA* (protochlorophyllide reductase, chloroplastic), which is involved in chlorophyll biosynthesis by catalyzing the formation of chlorophyllide from protochlorophyllide during biosyntheses of chlorophylls and bacteriochlorophylls.

### Management of proteins

External stresses disturb the process of protein synthesis and denaturation. Cells deal with denatured proteins via either rescue or degradation. Cells produce heat shock proteins, which act as molecular chaperones to rescue and stabilize misfolded proteins pathway [16]. Therefore, heat shock protein is sometimes considered as an indicator of stress level. Here we found that heat shock protein DEGs were all downregulated under shade, suggesting that plants became less stressed under shade, which supports the conclusion from the photosynthesis pathway analysis. For proteins that are damaged and cannot be rescued, cells engage the process of protein degradation through the ubiquitin/proteasome pathway [17]. Similar to previous studies [18, 19], three of the four ubiquitination-related DEGs were downregulated, suggesting a decreased need for disposal of denatured proteins under reduced light conditions. Gene expression patterns of protein rescue and degradation indicate that protein synthesis is more disrupted in albino leaves, which may have caused the accumulation of total amino acids during the albinism process [8]. Indeed, we have identified that DEGs are involved in the metabolism of cysteine, methionine, lysine, glycine, serine, threonine and tyrosine.

### Oxidation stress reduction

Reactive oxygen species (ROS) is a group of molecules produced from metabolic processes in chloroplast, mitochondria and peroxisome. ROS include superoxide anion, hydrogen peroxide, and hydroxyl radicals, which are all highly reactive molecules and affect normal cellular functions by interacting with nucleic acids, proteins and lipids [20]. ROS is normally controlled within a safe range through detoxification processes, but can be trigger by environmental stresses, e.g., strong light condition [21, 22]. On the one hand, ROS causes molecular damages and affects cellular processes [20, 21]; on the other hand, the effect of ROS might be a strategy for cells to survive stressful conditions by temporarily turning off some processes [22]. For example, in Chinese poplar *Populus simonii*, ROS production in the early chilling response resulting in inhibition of photosynthesis to produce a survival advantage [23]. Cytochrome P450 catalyzes most of the steps in the detoxification process of secondary metabolisms in plants [24]. In this study, we identified nine transcripts from Cytochrome P450 were significantly more expressed in plants under shade than that in plants exposed to natural sunlight. Similar results were also discovered for other enzymes involved in the oxidation-reduction process, including flavonoid 3′-hydroxylase 2, flavonoid 3′-hydroxylase 3, leucoanthocyanidin dioxygenase, carbamoyl-phosphate synthetase 2, aspartate transcarbamylase, and dihydroorotase. Therefore, the albino leaves might be caused by the accumulation of ROS, which is a response of plants to deal with strong light conditions.

### Transcription factors

Transcription factors (TFs) regulate the complex transcription network and therefore are involved in a series of mechanisms to cope with abiotic stresses. We observed significant downregulation of TFs after shading, suggesting the reduction of environmental stress. The differentially expressed TFs in this study belong to gene families of *MYB*, *bHLH*, *Ethylene Response Factors (ERFs)*, *NAC*, *GRAS*, *WRKY*, etc. Regulation of these gene families has been previously shown responsive to abiotic stressors [25], such as temperature [26–28], chemicals [29, 30], salinity [31], light condition [9, 32]. It is not surprising that *NAC* and *WRKY* were both significantly regulated as they are two of the largest TF families in plants. In *C. sinensis*, *NAC* and *WRKY* have been shown to be responsive to all the aforementioned stressors [9, 28, 29, 31]. In *C. sinensis* ‘Shuchazao’, the expression of *MYB* family genes has been shown to be positively associated with the UV level [32]. Therefore, the upregulation of *MYB* family genes in the natural sunlight exposed group in this study may be a result of strong UV radiation. In addition, *MYB* TFs are involved in flavonoid biosynthesis [33], which has been shown to be significantly different between treatment groups in this study, e.g., catechins and polyphenols. However, the direct association warrants further investigation. Previous studies showed that *bHLH* TFs likely function cooperatively with *MYB*s to deal with environmental stressors and affect flavonoid biosynthesis [34]. The *ERFs* are involved in the ethylene signaling pathway that regulates many processes in development stress responses. We observed three downregulated genes (*CRF4*, *ERF4* and *RAP2–4*) and one up-regulated gene (*WIN1*).

## Conclusions

Taking together, tea is the most consumed non-alcoholic beverages around the world and new varieties are still continually being developed. Knowledge on the molecular basis of albinism provides valuable information that is commercially relevant. This paper provided some mechanistic understanding of albinism at multiple biological levels from transcriptome, molecular to cellular, which all suggest albinism in young leaves might be a result of stress responses. Further research in this area should lead to the accumulation of adequate information to allow a comprehensive understanding of how leaf color is affected by different environmental factors.

## Methods

### Plant and treatments

Twenty-year-old tea plants of *C. sinensis* cultivar ‘Huangjinju’ were grown at Eco-tea Garden of Jiangxi Sericulture and Tea Research Institute, Jiangxi province, China (28°22′14.5″N 116°00′05.8″E). Elevation of experiment field is 36 m. Abiotic and biotic conditions, except the light condition, were maintained the same throughout this study. On May 10, 2018, tea plants were trimmed and divided into two groups: one group was under natural sunlight, the other group was covered with black polyethylene shading net (width,15.0 m; length 6.0 m; height: 1.8 m) that blocks out 70% of sunlight. On June 10, 2018 when plants reached one bud and two leaves stage, the difference in leave color was visually apparent. Leaf samples were randomly sampled and snap-frozen in liquid nitrogen before stored at − 80 °C.

### Determination of pigmentation content

Pigmentation content in leaves was quantified using two methods: Soil-Plant Analyses Development (SPAD) and high-performance liquid chromatography (HPLC). The SPAD method is a quick measurement of relative chlorophyll amount in fresh leaf samples using a hand-held device SPAD-502PLUS (Spectrum Technologies, Konica Minolta, Japan). This SPAD device takes readings directly from fresh leaves and therefore, does not require additional sample processing. The SPAD measurements were conducted on six biological replicates for each treatment. In each replicate, a total of ten leaves were measured and the average value was logger as a SPAD value. The HPLC analysis of pigments including chlorophyll a, chlorophyll b, and b-carotene was previously described in Li et al., 2015 [6]. Briefly, 100 randomly harvested fresh young shoots were ground, and 0.2 g of the ground product was extracted with 10 ml acetone and 0.1 g polyvinylpolypyrrolidone. The mixture was centrifuged at 12,000 rpm for 15 min (4 °C). The supernatant was analyzed on the LC-20AT HPLC System (Shimadzu, Kyoto, Japan) with a TC-C18 column (Agilent Technologies Inc., Santa Clara, CA, USA). The injection volume was 20 μl. The column was eluted at 35 °C with a linear gradient increasing from 80 to 100% mobile phase B (acetonitrile/methanol/chloroform:15/4/1, v/v/v) over 20 min at a flow rate of 1 ml min^− 1^. After an additional 15 min at 100% mobile phase B, the gradient was linearly decreased from 100 to 80% over 5 min, and then 80% mobile phase B for an additional 5 min. The eluent was detected at the wavelength of 440 nm. An external standard was used as an authentic reference to quantify detected pigments.

### Determination of free amino acids content

Free amino acid content was determined according to the national standard (GB/T 8314–2013). Briefly, fresh shoots (one bud and two leave stage) were fixed by steaming for three min. Fixed leaves were then dried at 80 °C for 3 h. Dried leaves were ground to powder and passed through a 0.45 mm-mesh sieve. Then, 3.0 g of the fine power were placed in 450 ml of boiling water for 45 min to make the extract solution. The extract solution was then filtered through a Double-Ring No. 102 filter paper (Xinhua Paper Industry Co. Ltd., Hangzhou, China), and the volume was increased to 500 ml by adding distilled water. Next, 1 ml of the solution was transferred to a 25 ml flask, followed by the addition of 0.5 ml of buffer (pH 8.0) containing 63 mM Na_2_HPO_4_ and 3 mM KH_2_PO_4_, 0.5 ml of a 2% ninhydrin solution (2 g ninhydrin and 80 mg SnCl_2_·2H_2_O dissolved in 100 ml of water). The flask was incubated at boiling temperature for 15 min. The volume was then increased to 25 ml with H_2_O. The absorbance (570 nm) of the mixture was measured with a UV Spectrophotometer U-2800 (Hitachi High-Technologies Corporation, Tokyo, Japan). Total free amino acid content was calculated from a standard curve generated with varying concentrations of glutamine.

### Determination of total polyphenols, catechins and caffeine content

The concentration of total polyphenols was determined by spectrophotometry, as described in the national standard of China (GB/T 8313–2018) with minor modifications. Briefly, 100 randomly harvested fresh young shoots (one-bud and two-leaf stage) were ground, and 0.2 g of the ground product was mixed with 5.0 mL 70% ethanol. The mixture was incubated at 70 °C for 10 min and stirred every 5 min. After the mixture was cooled to room temperature, it was centrifuged at 3500 rpm for 10 min. The supernatant was separated and added to a 10 mL volumetric flask. The sediment was remixed with 5.0 mL 70% ethanol and repeated the procedure. Supernatants from each step were collected in a volumetric flask and adjusted to 10 mL with deionized water (4 °C) to form the extract solution. A volume of 1 mL of extract solution was placed in a measuring flask and adjusted to 100 mL with deionized water to form the test solution. To measure total polyphenols, test solution (1 mL) was then mixed with 10% Folin Ciocalteu reagent (5 mL) in a test tube for 5 min. Next, 4 mL of Na_2_CO_3_ solution (75 g L^− 1^) was added to the test tube, and the mixture was stirred for one h at room temperature. The absorbance of test solution was measured with a UV Spectrophotometer U-2800 (Hitachi High-Technologies Corporation, Tokyo, Japan) at 765 nm. Gallic acid at different concentrations (10, 20, 30, 40, and 50 μg mL^− 1^) was used as a reference, and the polyphenol results were presented as gallic acid equivalent concentrations. To measure catechins and caffeine, test solution (2 mL) was first mixed with 25 mL stabilizing solution (25 ml EDTA-2Na at 10 mg mL^− 1^; 50 mL chromatographically pure Acetonitrile; 25 mL ascorbic acid at 10 mg mL^− 1^: water, 400 ml) and then filtered through a 0.45 μm membrane (Millipore, Billerica, MA, USA). An aliquot of 10 μL filtrate was measured using an LC-10ATVP HPLC system (Shimadzu, Tokyo, Japan). The HPLC conditions were as follows: inject volume, 20 μl; C18 column, 5 μm, 250 mm × 4.6 mm (Agilent Technologies Inc., Santa Clara, CA, USA); 35 °C; gradient elution: started with phase A (100%) for 10 min, in 15 min phase A decreased to 68% phase A and 32% phase B and held for 10 min, reaching 100% phase A; flow rate: 1 ml min^− 1^; mobile phase A (2 ml EDTA-2Na at 10 mg mL^− 1^; 90 mL chromatographically pure Acetonitrile; 25 mL Acetic acid at 20 mg mL^− 1^: water, 888 ml), mobile phase B (2 ml EDTA-2Na at 10 mg mL^− 1^; 800 mL chromatographically pure Acetonitrile; 20 mL Acetic acid at 10 mg mL^− 1^: water, 178 ml); detection wavelength: 278 nm.

### Chloroplast ultrastructure analysis

The chloroplast ultrastructure was analyzed using transmission electron microscopy (TEM) facilities at the Institute of Virology, Chinese Academy of Science, Jiangxi Province, China, following the protocol previously described in Wang et al., 2014 [35]. Briefly, fresh leaves were firstly cut into 1 cm × 2 cm pieces. Pieces without leaf veins were chosen and fixed in glutaraldehyde solution (2.5%) at 4 °C overnight. Samples were then washed with phosphate buffer (0.1 M, pH 7.0) three times (20 min each). After washing, samples were fixed again in O_S_O_4_ solution (1%, 4 °C) for 2–3 h and washed three times with phosphate buffer (0.1 M, pH 7.0). After fixation steps, samples were sequentially subjected to a graded ethanol series (50, 70, 80, 85, 90, 95 and 100%) for dehydration. Each dehydration step lasted 15 min, followed by soaking in 100% ethanol for 20 min. Dehydrated samples were sequentially drenched in the mixture of acetone: epoxy resin (2:1), acetone: epoxy resin (1:1) and epoxy resin. Drenched samples were embedded in pure epoxy resin at 60 °C for 48 h. After embedding, 60–100 nm thick sections were cut with an EM UC6 microtome (Leica, Vienna, Austria) and stained with saturated uranyl acetate in 50% ethanol for 15 min and 0.2% (w/v) lead citrate for 15 min. Images were taken under a Tecnai G^2^ 20 TWIN transmission electron microscope (FEI, Oregon, United States).

### RNA-Seq and bioinformatics

Total RNA was extracted using a TRlzol reagent (Invitrogen™ Life Technologies, CA, USA) according to the manufacturer’s manual. The RNA concentration was quantified using Qubit® RNA Assay Kit in Qubit® 2.0 Flurometer (Life Technologies, CA, USA) while RNA integrity was tested with RNA Nano 6000 Assay Kit of the 2100 Bioanalyzer Instrument (Agilent Technologies, CA, USA). RNA-Seq libraries were prepared using NEBNext® Ultra™ RNA Library Prep Kit (NEB, USA) according to the manufacturer’s manual. Libraries were pair-end sequenced on an Illumina Hiseq 2500 platform (Novogene, Beijing, China). Raw reads were quality controlled by filtering adapter sequences, reads containing more than 10% ploy-N and low-quality sequences using customized Perl script. Clean reads were aligned to tea plant *Camellia sinensis* reference genome [36] (http://tpia.teaplant.org/) using Hisat2 v2.0.4 [37]. HTSeq v0.9.1 was used to count the reads numbers mapped to each gene.

### Differential gene expression

Gene counts were normalized to FPKM (Fragments Per Kilobase of transcript sequence per Millions base pairs sequenced) based on the length of the gene and number of reads mapped to this gene [38]. Differentially expressed gene (DEGs) analysis of two light conditions (three biological replicates per condition) was performed using the R package DESeq (1.18.0) [39]. DESeq determines differential gene expression using statistical methods modelled with the negative binomial distribution. A Benjamin -Hochberg’s approach was used to adjust the *p*-values with cutoff at 0.05 [40].

### Enrichment analysis of DEGs

Functional category and pathways of DEGs were annotated by searching against public databases to form a better understanding of the molecular mechanism. Gene Ontology (GO) enrichment analysis of DEGs was implemented by the GOseq R package [41], in which gene length bias was corrected. The significance level of enrichment analysis was tested using Fisher’s Exact test. GO terms with over-represent p-value less than 0.05 were considered significantly enriched by DEGs. Kyoto Encyclopedia of Genes and Genome (KEGG) pathway database is a collection of pathway maps representing current knowledge on molecular interaction and reaction networks [42]. Transcripts were aligned against the KEGG database for ortholog annotation. Statistical enrichment of pathways was tested in KOBAS software [43].

### Statistical analysis

Significant differences between treatments were determined using a Student’s two-tailed t-test with SPSS 20.0 software (IBM Corporation, Chicago, IL, USA) and values of *p* < 0.05 were considered statistically significant.

## Supplementary information

**Additional file 1: Table S1**. Summary of differentially expressed genes

**Additional file 2: Table S2.** Summary of gene ontology (GO) analyses.

**Additional file 3: Table S3**. Summary of Kyoto Encyclopedia of Genes and Genomes (KEGG) pathway enrichment analyses.

**Additional file 4: Fig. S1.** Gene ontology enrichment of differentially expressed genes identified in leaves under direct sunlight versus under shade. Only up to 10 terms are selected to present. Horizontal redline represents the threshold of significance after false discovery rate correction.

## Data Availability

Raw sequencing data files are available in the NCBI SRA database with project accession NO. PRJNA589096.

## Abbreviations

DEG: Differentially expressed gene
GO: Gene ontology
Gr: grana
HPLC: high performance liquid chromatography
S: natural sunlight
KEGG: Kyoto Encyclopedia of Genes and Genomes
OG: osmiophilic granules
ROS: Reactive oxygen species
SPAD: Soil-Plant Analyses Development
SC: shade control
SG: starch granules
TEM: transmission electron microscopy
Th: thylakoids
