# Supplementary material for: Transcriptomic analysis reveals mechanism of light-sensitive albinism in tea plant Camellia sinensis ‘Huangjinju’
Source: BMC Plant Biol. 2020 May 14;20:216. doi: 10.1186/s12870-020-02425-0 (PMC7227349; doi:10.1186/s12870-020-02425-0)
Supplement: Supplementary file 4 — Additional file 4: Fig. S1. Gene ontology enrichment of differentially expressed genes identified in leaves under direct sunlight versus under shade. Only up to 10 terms are selected to present. Horizontal redline represents the threshold of significance after false discovery rate correction. [file 12870_2020_2425_MOESM4_ESM.docx]

Supplementary figures


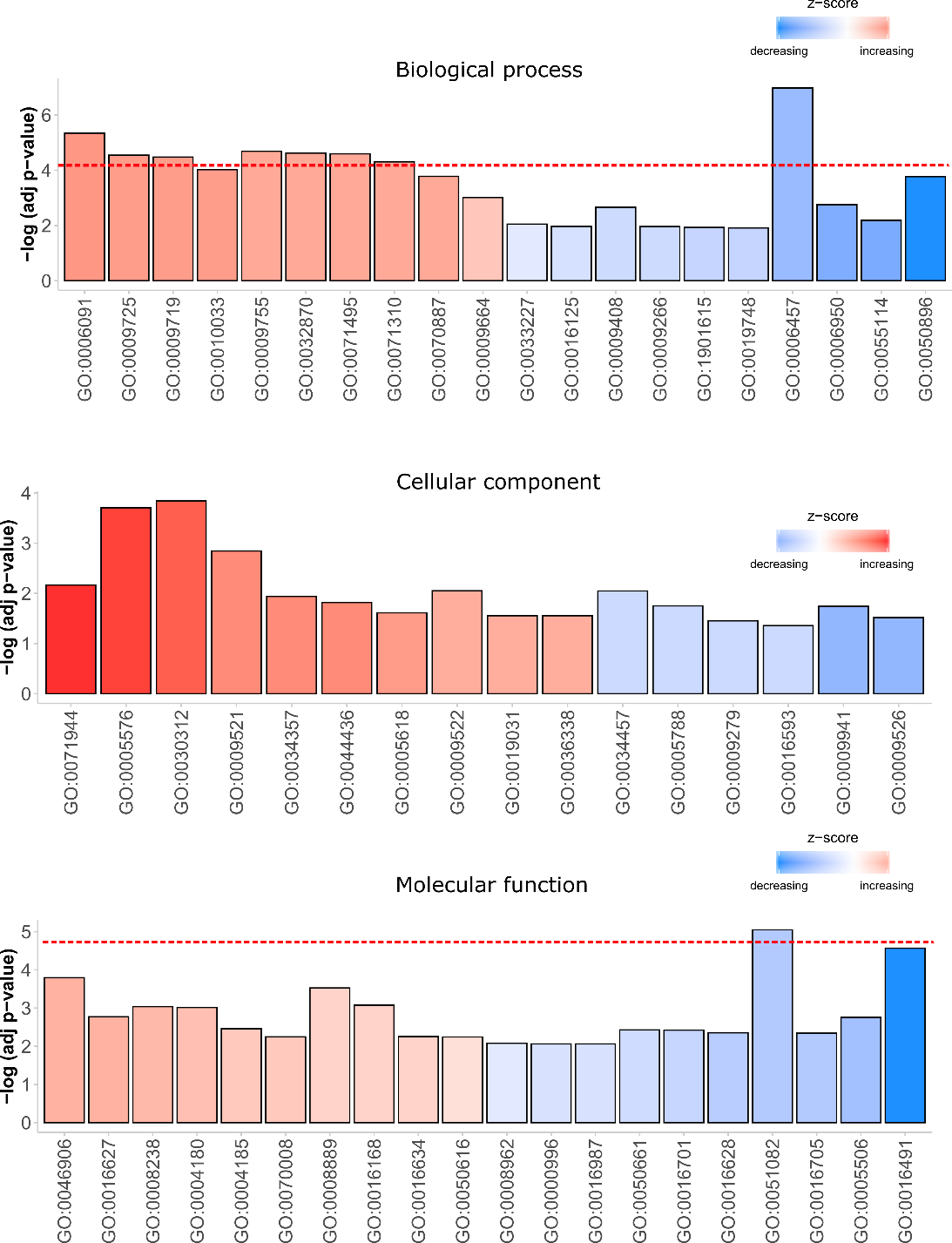


**Fig. S1 Gene ontology enrichment of differentially expressed genes identified in leaves under direct sunlight versus under shade.** Only up to 10 terms are selected to present. Horizontal redline represents the threshold of significance after false discovery rate correction.
